# MicroRNAs: Potential Biomarkers and Therapeutic Targets for Alveolar Bone Loss in Periodontal Disease

**DOI:** 10.3390/ijms17081317

**Published:** 2016-08-11

**Authors:** Tadayoshi Kagiya

**Affiliations:** Division of Functional Morphology, Department of Anatomy, Iwate Medical University, 2-1-1 Nishitokuta, Yahaba-cho, Iwate 028-3694, Japan; tkagiya@iwate-med.ac.jp; Tel.: +81-19-651-5111; Fax: +81-19-908-8010

**Keywords:** alveolar bone loss, exosomes, extracellular vesicles, microRNAs, osteoclasts, periodontal disease

## Abstract

Periodontal disease is an inflammatory disease caused by bacterial infection of tooth-supporting structures, which results in the destruction of alveolar bone. Osteoclasts play a central role in bone destruction. Osteoclasts are tartrate-resistant acid phosphatase (TRAP)-positive multinucleated giant cells derived from hematopoietic stem cells. Recently, we and other researchers revealed that microRNAs are involved in osteoclast differentiation. MicroRNAs are novel, single-stranded, non-coding, small (20–22 nucleotides) RNAs that act in a sequence-specific manner to regulate gene expression at the post-transcriptional level through cleavage or translational repression of their target mRNAs. They regulate various biological activities such as cellular differentiation, apoptosis, cancer development, and inflammatory responses. In this review, the roles of microRNAs in osteoclast differentiation and function during alveolar bone destruction in periodontal disease are described.

## 1. Introduction

**Figure 1:**
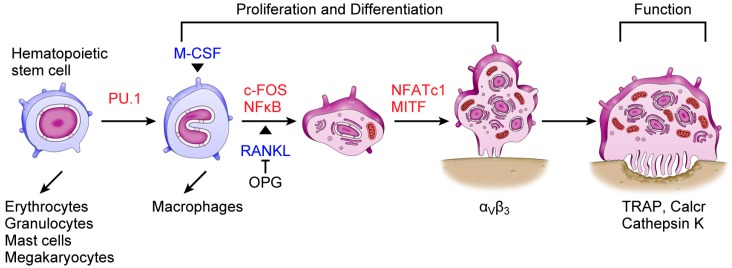
Schematic view of osteoclast differentiation. Osteoclasts are derived from hematopoietic stem cells. Cytokines macrophage colony-stimulating factor (M-CSF) and receptor activator of nuclear factor κB ligand (RANKL) are essential for osteoclastogenesis. Binding of RANKL to its receptor, receptor activator of nuclear factor κB (RANK), activates nuclear factor of activated T cells, cytoplasmic 1 (NFATc1), which is a master regulator of osteoclastogenesis. NFATc1 works with other transcription factors, such as activator protein-1 (AP-1), PU.1, and microphthalmia-associated transcription factor (MITF) to induce various osteoclast-specific genes, such as TRAP, Calcr, and Cathepsin K. RANK-RANKL interaction is inhibited by the decoy receptor osteoprotegerin (OPG) expressed by stromal cells and osteoblasts. Blue, cytokines essential for osteoclastogenesis; red, transcription factors.

**Figure 2:**
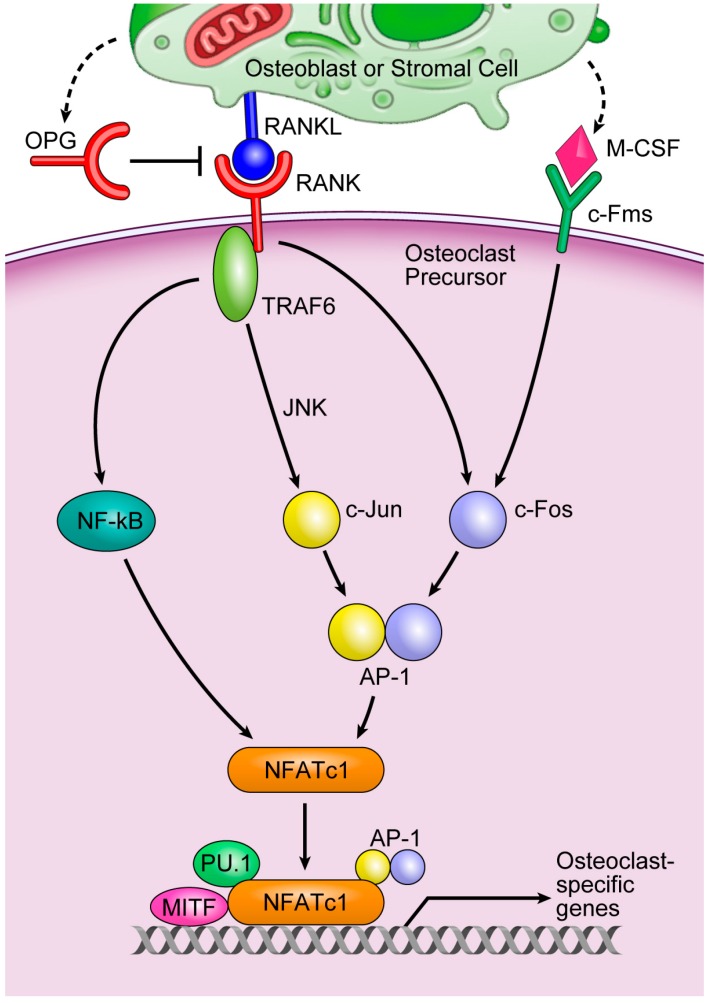
A key osteoclastogenesis signaling cascade. Cited from [2]. The binding of M-CSF to its receptor, c-Fms, induces the transcription factor c-Fos, whereas the binding of RANKL to its receptor, RANK, leads to the recruitment of TNF-receptor-associated factor 6 (TRAF6), the main adapter molecule of RANK. TRAF6 activates nuclear factor κB (NF-κB) and mitogen-activated kinases including c-Jun N-terminal kinase (JNK). JNK activates the transcription factor c-Jun. RANKL/RANK also induces c-Fos to form AP-1, a heterodimeric transcription factor, with c-Jun. AP-1 and NF-κB then induce NFATc1, a master transcription factor that regulates osteoclast differentiation. NFATc1 works with other transcription factors, such as AP-1, PU.1, and microphthalmia-associated transcription factor (MITF) to induce various osteoclast-specific genes.

Periodontal disease is one of the most common oral diseases worldwide. It is an inflammatory disease caused by bacterial infection of tooth-supporting structures that causes the destruction of alveolar bone [1]. Bone mass is determined by the balance between osteoblastic bone formation and osteoclastic bone resorption. Although bone formation and bone resorption are balanced under normal physiological conditions, excessive bone resorption occurs in periodontal disease, which results in the destruction of alveolar bone. The only cells that resorb bone are osteoclasts. Osteoclasts are large, tartrate-resistant acid phosphatase (TRAP)-positive multinucleated cells derived from hematopoietic stem cells (Figure 1) [1,2]. Osteoclast differentiation is controlled by a variety of hormones, growth factors, and cytokines. Among them, receptor activator of nuclear factor κB ligand (RANKL), which is expressed in stromal cells, osteoblasts, and T cells, is essential for osteoclast differentiation [1]. The binding of RANKL to its receptor, receptor activator of nuclear factor κB (RANK), finally activates nuclear factor of activated T cells, cytoplasmic 1 (NFATc1), which is a key regulator of osteoclast differentiation. NFATc1 works with other transcription factors such as activator protein-1 (AP-1), PU.1, and microphthalmia-associated transcription factor (MITF) to induce various osteoclast-specific genes (Figure 2) [2,3].

Since the discovery of the first microRNA (miRNA) in *Caenorhabditis elegans* in 1993 [4,5], RNA biology has advanced greatly. miRNAs are small, endogenous, non-coding RNAs approximately 20–22 nucleotides in length. They act in a sequence-specific manner to regulate gene expression at the post-transcriptional level through cleavage or translational repression of their target mRNAs [1,2,6]. To date, 2588 miRNAs have been identified in humans (miRBase database, http://www.mirbase.org/). miRNAs participate in the regulation of several biological activities such as cellular differentiation, apoptosis, cancer development, and inflammatory responses. Recently, the involvement of miRNAs in periodontal disease has been reported [1,7,8,9,10,11]. Focusing on alveolar bone loss in periodontal disease, this paper describes the roles of miRNAs in osteoclast differentiation and function.

## 2. Biogenesis of MicroRNAs (miRNAs)

**Figure 3:**
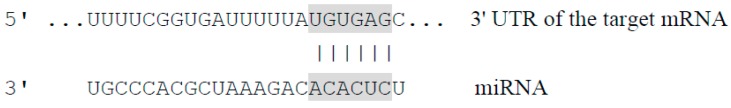
Binding of the microRNA (miRNA) seed region to its complementary site within the target mRNA. The miRNA sequence typically located from nucleotides 2 to 7 at the 5’ end is termed the seed region. This region binds to its complementary site within the 3’-untranslated region (UTR) of the target mRNA. Although a perfect match is not required for base pairing between the miRNA and its target mRNA, binding at the seed region must be perfectly complementary.

miRNA is either transcribed from its own promoter in an intergenic region or is processed from the intronic region of a coding gene as a long primary transcript, known as pri-miRNA. This pri-miRNA is processed into a 70–100 nucleotide precursor miRNA (pre-miRNA) by the RNase III enzyme Drosha and its co-factor DGCR8 in the nucleus. The RNA is then exported to the cytoplasm by a transport protein, Exportin-5. In the cytoplasm, it is further processed by another RNase III enzyme, Dicer. Thus, pre-miRNA is cleaved into a mature miRNA duplex. The resulting single-stranded mature miRNAs are ultimately incorporated into an RNA-induced silencing complex (RISC) that contains argonaute (Ago) family proteins [2,6,12,13]. miRNAs regulate gene expression by binding to mRNA. The selectivity of miRNA action is conferred mainly via nucleotides 2–7 located at the 5’ end, termed the “seed region”, which pairs to its complementary site in the 3’-untranslated region (UTR) of the target mRNA [14]. Although a perfect match is not required for base-pairing of the miRNA to its target mRNA, the seed region must be perfectly complementary (Figure 3). Thus, the RISC inhibits the translation of or degrades the target mRNAs.

## 3. Osteoclasts and miRNAs

Recent studies have revealed that miRNAs play important roles in osteoclast differentiation and function [6]. We reported that the expression of 52 mature miRNAs differed more than two-fold between untreated cells and cells treated with RANKL during osteoclastogenesis [1]. Table 1 lists the miRNAs that have been implicated in periodontal disease-related osteoclastogenesis. This section discusses selected important miRNAs.

miR-21 is highly expressed not only in the gingiva during periodontitis (Table 1) but also in cells during osteoclastogenesis [1]. Some critical pathogenic factors in periodontal disease induce miR-21 expression. Lipopolysaccharide (LPS) is a major pathogenic component of the cell wall of Gram-negative bacteria and an important factor contributing to periodontal disease. LPS signaling is mediated by Toll-like receptors leading to nuclear factor κB (NF-κB) activation [15]. In macrophages, LPS promotes NF-κB activation and decreases programmed cell death 4 (PDCD4) protein levels via miR-21 induction [16]. RANKL-induced c-Fos also upregulates miR-21 gene expression, which downregulates the expression of PDCD4, a negative regulator of osteoclastogenesis [17]. Tumor necrosis factor-α (TNF-α), which is present at high levels in both gingival crevicular fluid and periodontal tissues of diseased sites, is involved in the pathogenesis of periodontitis [1]. TNF-α acts through several pathways including NF-κB, which is involved in inflammation and apoptosis [18]. miR-21 is an NF-κB transactivational gene, and the combination of TNF-α and RANKL treatment increases miR-21 expression compared with RANKL treatment alone during osteoclast differentiation [1].

The miR-29 family includes miR-29a, miR-29b, and miR-29c, which are overexpressed in gingiva during periodontitis (Table 1). miR-29 plays critical roles in bone tissues as well as in the gingiva [6,8,10]. miR-29a and miR-29c positively regulate osteoblast differentiation by controlling the expression of osteonectin [19]. Canonical Wnt signaling, which is activated during osteoblast differentiation, induces miR-29 expression [19]. miR-29b also promotes osteogenesis by directly downregulating inhibitors of osteoblast differentiation [20]. miR-29b is induced by NF-κB and is upregulated in cells treated with TNF-α/RANKL relative to RANKL-treated cells during osteoclastogenesis [1,21]. Rossi et al. [22] reported that miR-29b expression decreases progressively during human osteoclast differentiation. Ectopic miR-29b expression suppresses c-Fos and matrix metalloproteinase 2 (MMP-2) expression and inhibits osteoclast formation. By contrast, Franceshetti et al. [23] reported that miR-29 family members are positive regulators of osteoclast differentiation. The expression of all miR-29 family members increases during osteoclast differentiation. Knockdown of miR-29 causes impaired osteoclastic commitment and migration of pre-osteoclasts without affecting cell viability, actin ring formation, or apoptosis in mature osteoclasts [6,23]. Furthermore, miR-29 directly targets cell division control protein 42 (Cdc42), SLIT-ROBO Rho GTPase-activating protein 2 (Srgap2), G protein-coupled receptor 85 (Gpr85), nuclear factor I/A (NFI-A), CD93, and calcitonin receptor (Calcr), which are important for osteoclast differentiation and function [23]. In summary, miR-29 is considered to play a critical role in osteoclast differentiation and function despite conflicting reports.

**Figure 4:**
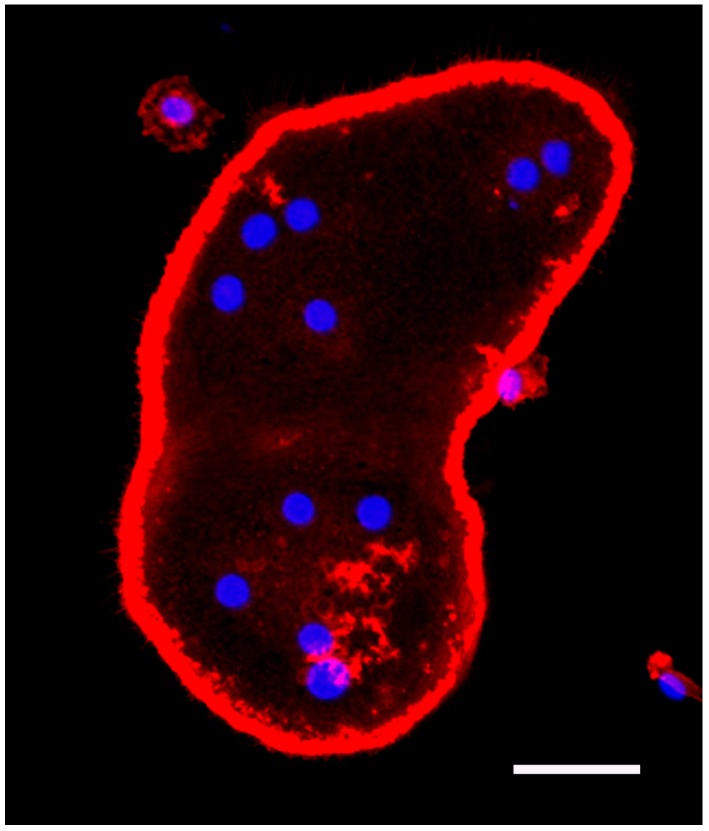
Microscopic image of a cultured osteoclast. Murine bone marrow macrophages were incubated for 82 h with RANKL (100 ng/mL) and M-CSF (10 ng/mL), resulting in osteoclast formation. After culturing, cells were fixed with 4% paraformaldehyde in phosphate buffer and stained with DAPI and Rhodamine B-conjugated phalloidin. Osteoclasts are multinucleated giant cells with a ring-shaped osteoclast-specific podosome belt termed the actin ring. Blue, nuclei; red, actin; scale bar = 50 µm.

miR-31 is expressed in a wide variety of tissues and cells [6,24]. The expression of miR-31 is decreased in gingiva with periodontitis compared to healthy gingiva (Table 1). miR-31 plays a critical role in osteoclastic bone resorption. The activated osteoclast has a ring-shaped osteoclast-specific podosome belt called the “actin ring” (Figure 4). The actin ring forms a sealing zone where the osteoclast adheres tightly to the bone surface [6,25]. miR-31 is one of the highly upregulated miRNAs during osteoclast development. Inhibition of miR-31 suppresses osteoclast formation and bone resorption. miR-31 controls the cytoskeleton in osteoclasts by regulating the expression of RhoA, which regulates the formation of the actin ring [26].

miR-34a was recently shown to be involved in osteoclast and osteoblast differentiation. It is reported that miR-34a blocks osteoporosis and bone metastasis by inhibiting osteoclast differentiation [27]. The expression level of miR-34a decreases during osteoclastogenesis and knockdown of miR-34a promotes osteoclast differentiation, while overexpression of miR-34a impairs this differentiation [6,27]. In contrast, osteoblast differentiation is inhibited in miR-34a knockout mice but is promoted in osteoblastic miR-34a conditional transgenic mice. Krzezinski et al. [27] identified transforming growth factor-β-induced factor 2 (Tgif2) as a direct target of miR-34a. Tgif2 is an essential factor for osteoclast differentiation, and NFATc1 and AP-1 induce Tgif2 expression during osteoclastogenesis [27].

Although miR-124 is highly abundant in the brain and contributes to the differentiation of neural progenitors into mature neurons [6,28], it also plays an important role in osteoclast formation. miR-124 expression decreases in a time-dependent manner during murine osteoclast differentiation [29]. Inhibition of miR-124 enhances osteoclastogenesis and the expression of NFATc1. Ectopic miR-124 expression inhibits osteoclast differentiation and NFATc1 expression without affecting the expression of the NF-κB p65 subunit or c-Fos [29]. These results indicate that miR-124 may directly regulate NFATc1 expression. Indeed, Nakamachi et al. [28] demonstrated that NFATc1 is a direct target of miR-124 in human osteoclasts. miR-124 inhibits the progression of adjuvant-induced arthritis in a rat model of rheumatoid arthritis (RA) by reducing osteoclast formation. RA and periodontitis share a similar pathophysiology, characterized by destructive inflammation that culminates in localized bone loss. Considering this similarity, miR-124 may play a critical role in alveolar bone loss in periodontal disease.

miR-125a expression is increased in gingiva with periodontitis compared to healthy gingiva (Table 1), and it is also involved in osteoclastogenesis. We reported that treatment of RAW264.7 cells with TNF-α/RANKL and RANKL triggers time-dependent upregulation of miR-125a expression during murine osteoclast differentiation [1]. De la Rica et al. [30] reported that two miRNA clusters, miR-212/132 and miR-99b/let-7e/125a, display rapid upregulation during human osteoclast differentiation. These miRNAs are activated directly by NF-κB, and their inhibition impairs osteoclast formation; however, Guo et al. [31] reported that miR-125a expression is dramatically downregulated during human osteoclast formation caused by M-CSF/RANKL treatment. Ectopic miR-125a expression impairs osteoclastogenesis, while its inhibition has the opposite effect. TNF-receptor-associated factor 6 (TRAF6), the main adapter molecule of RANK, was reported to be a direct target of miR-125a. In addition, Guo et al. [31] reported that NFATc1 binds to the promoter of miR-125a and inhibits transcription of miR-125a. Taken together, miR-125a plays a critical role in osteoclast differentiation, although reports differ on the exact mechanism of action.

miR-146a is an NF-κB-dependent gene that plays an important role in innate immunity [6,21,32]. For example, LPS rapidly induces miR-146a through the Toll-like receptor/NF-κB pathway in human macrophage-like cells [21]. Mature miR-146a is highly expressed in gingiva with periodontitis (Table 1). We reported that miR-146a expression increases during TNF-α-regulated osteoclast differentiation [1]; however, miR-146a overexpression inhibits osteoclast formation [33]. TRAF6 is a direct target gene of miR-146a [32]. Without TNF-α, the time-dependent miR-146a expression decreases in RAW264.7 cells during osteoclastogenesis [1]. Furthermore, overexpression of miR-146a does not affect proinflammatory cytokine production in human macrophage-like cells [34]. These reports suggest that miR-146a alone does not induce proinflammatory cytokine production in macrophages, while proinflammatory cytokines, such as TNF-α and LPS, promote miR-146a expression. Collectively, although miR-146a plays important roles in inflammatory responses, excessive miR-146a expression may serve as a negative feedback regulator of osteoclastogenesis.

**Figure 5:**
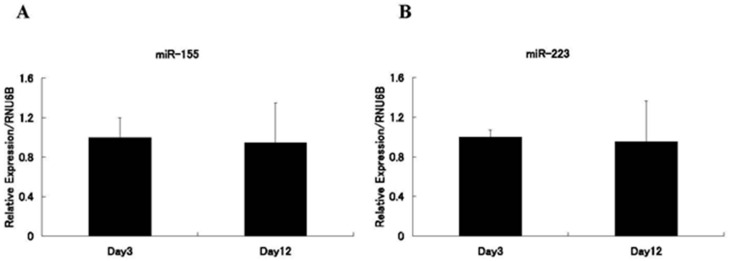
Expression levels of miR-155 and miR-223 during human osteoclast differentiation. Human peripheral blood CD14^+^ cells were treated with M-CSF (50 ng/mL) for three days, followed by RANKL (50 ng/mL) and M-CSF (25 ng/mL) for an additional nine days. Total RNAs were harvested at day 3 (macrophages) or day 12 (osteoclasts). The expression levels of miR-155 (**A**) and miR-223 (**B**) were then analyzed by quantitative RT-PCR. Values for each miRNA are expressed relative to those on day 3, which were set to 1. Quantification was performed using RNU6B as an endogenous control. The experiments were performed in triplicate. Data are presented as means ± SD. Student’s *t*-tests were performed to assess significant differences.

miR-155 is an inflammation-associated miRNA that regulates inflammation and immune cell function at multiple levels [6,35]. For example, leukotriene B_4_ (LTB_4_) is a lipid mediator formed from arachidonic acid and is one of the most potent stimulants of macrophages. LTB_4_ levels in gingival crevicular fluid correlate with periodontitis severity [36]. LTB_4_ enhances the generation of miR-155 to promote MyD88-dependent macrophage activation [37]. We reported that the expression level of miR-155 in murine bone marrow macrophages (BMMs) is upregulated by TNF-α/RANKL/M-CSF treatment, but is modestly downregulated by RANKL/M-CSF treatment during osteoclast formation [1]. Mann et al. [38] reported that miR-155 expression decreases during osteoclast differentiation. Our findings in BMMs are compatible with these reports; however, miR-155 expression was not significantly different between M-CSF-treated and RANKL/M-CSF-treated human peripheral blood CD14^+^ cells during osteoclastogenesis (Figure 5A). Mizoguchi et al. [39] reported that miR-155 levels in BMMs from wild-type mice are not significantly altered by RANKL treatment. Fewer osteoclasts were generated in vitro from BMMs of miR-155-deficient mice than from those of wild-type mice [40]. By contrast, Mann et al. [38] reported that ectopic miR-155 expression inhibits osteoclast formation by repressing MITF and PU.1, which are transcription factors important for osteoclast differentiation. Taken together, these reports suggest that miR-155 plays a critical role in osteoclast differentiation and that its downregulation may not be necessary for osteoclastogenesis [1,6].

Although miR-223 expression is increased in gingiva with periodontitis compared with healthy gingiva (Table 1), miR-223 is expressed in human monocytes, granulocytes, and platelets [1,6], and it is a central modulator of myeloid differentiation. Osteoclasts are hematopoietic stem cell-derived cells of the monocyte/macrophage lineage, and miR-223 plays a crucial role in osteoclast differentiation [6]. We and other researchers have observed that miR-223 expression decreases during murine osteoclastogenesis [1,41]. miR-223 regulates NFI-A and the expression of the c-Fms, M-CSF receptor, which is critical for osteoclast differentiation and function [42]. Ectopic miR-223 expression inhibits murine osteoclastogenesis, while its inhibition has the opposite effect [41]. However, miR-223 expression was not significantly different during human osteoclast differentiation (Figure 5B). Moutoula et al. [43] reported that overexpression of miR-223 promotes human osteoclastogenesis, whereas its inhibition has the opposite effect. In summary, although the mechanism of miR-223 action may differ between human and murine osteoclastogenesis, miR-223 plays a critical role in osteoclast differentiation.

## 4. Extracellular miRNAs

Recently, miRNAs were reported to be present in body fluids such as saliva, serum, urine, and cerebrospinal fluid [49]. These extracellular miRNAs are considered potential biomarkers and therapeutic targets. They are divided into two populations: vesicle-associated and non-vesicle-associated forms [2,6,49]. In the vesicle-associated form, miRNAs are present in exosomes and microvesicles. In the non-vesicle-associated form, miRNAs are detected in complexes with Ago proteins, high-density lipoproteins, or other proteins [49,50]. Of these, the study of exosomes is an area of intense interest. Exosomes are a type of extracellular vesicle (EV). EVs are membranous vesicles naturally released by most cells and are divided into three main types: apoptotic bodies, microvesicles, and exosomes. Apoptotic bodies are released by apoptotic cells and are 800–5000 nm in diameter. Microvesicles are produced by budding directly from the plasma membrane and are 50–1000 nm in diameter. Exosomes are originated from endosomes and are 40–100 nm in diameter [2,6]. The current techniques are inadequate for collecting each type of EV [2,51,52], and a consensus has yet to be reached with regard to the nomenclature of exosomes and microvesicles [35,53]. Thus, this section does not use the term “exosomes” but rather “EVs”.

Recent studies have begun to uncover that EVs play a role in cell-to-cell communication by the transfer of miRNAs, mRNAs, proteins, and lipids to recipient cells [49,54,55,56]. The release of EVs depends on the cell type and biological condition [2,49,57]. Although studies have revealed that miRNAs play important roles in bone metabolism, including alveolar bone, whether osteoclasts secrete EVs containing miRNAs was unknown until recently. We examined eight miRNAs in EVs that seemed to be critical for osteoclast differentiation including let-7e, miR-21, miR-33, miR-155, miR-210, miR-223, miR-378, and miR-1224. Of these, the expression levels of miR-378, miR-21, and miR-210 were very high, whereas no significant expression of miR-33 or miR-1224 was detected [2,58]. These results indicate that osteoclasts release EVs containing specific miRNAs, but not the entire set of intracellular miRNAs. Among the miRNAs detected in EVs of osteoclasts, miR-378 was highly expressed in the supernatant of LPS-treated macrophage-like cells compared with that from non-LPS-treated macrophage-like cells [59]. miR-378 is also highly expressed in the serum of breast cancer patients with bone metastasis compared with that of healthy people [45]. Osteolytic bone metastasis, caused by excessive osteoclast activity, frequently occurs during the later stages of breast cancer [2]. Collectively, these reports support miR-378 as a candidate biomarker for alveolar bone loss in periodontal disease. 

Alexander et al. [60] reported that two critical miRNAs that regulate inflammation, miR-146a and miR-155, are released from dendritic cells within EVs and are taken up by recipient dendritic cells. They also reported that miR-146a within EVs inhibits LPS-induced inflammation in mice, while miR-155 within EVs promotes this. This report suggests that miRNAs within EVs can be transferred between immune cells in periodontal tissues. Periodontal disease is an infectious disease; serum miR-146a and miR-223 have been reported to be reduced significantly in septic patients compared with healthy controls [61]. As discussed above, these miRNAs are associated with alveolar bone loss in periodontal disease. Therefore, they may represent candidates for periodontal disease markers in serum and saliva.

## 5. Materials and Methods

### 5.1. Bone Marrow Macrophage Culture and Fluorescence Staining of Actin and Nuclei

All animal experiments were evaluated and approved by the Animal Use and Care Committee of Iwate Medical University (registration number: 17-0068, Morioka, Japan). Five-week-old male ddY mice were purchased from Japan SLC Inc. (Hamamatsu, Japan). The mice were sacrificed, and their femurs and tibias were removed and dissected free of adherent soft tissue. The ends of the bones were cut, and the marrow cells were collected as described previously [1]. Red blood cells were removed by treatment with phosphate-buffered saline (PBS) containing 10 mM Tris and 0.83% NH_4_Cl. After washing with α-minimum essential medium (α-MEM; Invitrogen, Fredrick, MD, USA), the cells were seeded at a density of 2 × 10^5^ cells/cm^2^ and cultured in α-MEM containing 10% fetal bovine serum (FBS; Moregate Biotech, Bulimba, Australia) and 10 ng/mL recombinant mouse macrophage colony-stimulating factor (M-CSF) (R&D Systems Inc., Minneapolis, MN, USA). After two days, the medium was changed, and the cells were cultured in the presence of M-CSF (10 ng/mL) and recombinant human soluble RANKL (PeproTech EC, London, UK) (100 ng/mL) for an additional three days. After culture, the cells were fixed with 4% paraformaldehyde in 0.1 M phosphate buffer (pH 7.4) for 10 min at room temperature. The samples were washed three times with PBS. The cells were incubated for 90 min at 37 °C with 0.5 mg/mL phalloidine-tetramethylrhodamine B isothiocyanate (Sigma-Aldrich, St. Louis, MO, USA). After washing three times with PBS, the cells were incubated for 10 min at room temperature with DAPI solution (diluted 1:1000; Dojindo, Kumamoto, Japan). The cells were then washed three times with PBS and observed using a confocal laser scanning microscope (LSM-510; Zeiss, Oberkochen, Germany).

### 5.2. Human Peripheral Blood CD14^+^ Cell Culture and Quantitative RT-PCR Analysis

Human peripheral blood CD14^+^ cells were purchased from Lonza (Basel, Switzerland). The cells were seeded at a density of 2 × 10^4^ cells/cm^2^ and cultured in α-MEM containing 10% FBS and 50 ng/mL recombinant human M-CSF (R&D Systems). After three days, the medium was changed, and the cells were cultured in the presence of M-CSF (25 ng/mL) and recombinant human soluble RANKL (50 ng/mL) for an additional nine days. The medium was exchanged every three days. To evaluate miRNA expression, total RNAs were harvested on days 3 or 12 using a mirVana™ miRNA Isolation Kit (Ambion, Austin, TX, USA). The RNA was reverse-transcribed using a TaqMan MicroRNA Reverse Transcription Kit (Applied Biosystems, Foster City, CA, USA). Expression of mature miRNAs was analyzed using appropriate TaqMan^®^ miRNA assays (Applied Biosystems) and Premix Ex Taq™ probe qPCR (Takara Bio, Otsu, Japan) according to the manufacturers’ protocols. Quantification was performed using RNU6B as an endogenous control. The 2^−ΔΔ*C*t^ method was used to calculate relative miRNA expression levels. 

## 6. Conclusions

Recent studies have demonstrated that miRNAs are involved in periodontal tissue homeostasis and pathology. Note that miRNAs highly expressed in periodontal disease gingiva are prone to being miRNAs that are important for osteoclast differentiation. Although whether gingival crevicular fluid contains miRNAs is unknown, miRNAs probably can be used as periodontal disease-specific biomarkers in saliva, serum, and gingival crevicular fluid. If EVs are transferred between cells in periodontal tissues, EVs may serve as therapeutic targets and can be used as drug delivery systems by packaging them with specific miRNAs, mRNAs, and proteins. Additional research is required to determine whether miRNAs can be used for periodontal disease treatment. We anticipate that this new therapeutic target for periodontal disease will open a new door in alveolar bone loss treatment.

## Figures and Tables

**Table 1:** Important miRNAs in periodontal disease-related osteoclastogenesis.

miRNA	Function(s)	Target(s)	Reference(s)	Gingiva with Periodontitis	Reference(s)
miR-21	P	Pdcd4, FasL	[17,44]	UP	[8]
miR-29b	N	C-FOS, MMP-2	[22]	UP	[8]
miR-29a/b/c	P	*Calcr*, *Cd93*, *Cdc42*, *Gpr85*, *Nfia*, *Srgap2*	[23]	UP	[8]
miR-31	P	RhoA	[26]	DOWN	[10]
miR-34a	N	*Tgif2*	[27]	UP/DOWN	[8,11]
miR-124	N	*NFATc1*	[28,29]	Not reported	
miR-125a	P/N	*TRAF6*, *TNFIP3*	[30,31]	UP	[8]
miR-141	N	*Mitf*, *Calcr*	[45]	DOWN	[10]
miR-146a	N	*TRAF6*	[32]	UP	[9]
miR-148a	P	*MAFB*	[46]	UP	[10]
miR-150	N	Opg	[47]	UP	[11]
miR-155	N	*Mitf*, *Socs1*, Pu.1	[1,38,48]	UP/DOWN	[9,10]
miR-223	P/N	NFI-A	[1,41,42,43]	UP	[10,11]

P, Positive regulator of osteoclastogenesis; N, Negative regulator of osteoclastogenesis.
